# Fabrication of Porous MXene/Cellulose Nanofibers Composite Membrane for Maximum Osmotic Energy Harvesting

**DOI:** 10.3390/ijms252313226

**Published:** 2024-12-09

**Authors:** Sha Wang, Zhe Sun, Mehraj Ahmad, Mengyu Miao

**Affiliations:** 1 Jiangsu Co-Innovation Center of Efficient Processing and Utilization of Forest Resources, Nanjing Forestry University, Nanjing 210037, China; 2 International Innovation Center for Forest Chemicals and Materials, Nanjing Forestry University, Nanjing 210037, China; 3 Department of Food Science and Engineering, College of Light Industry and Food, Nanjing Forestry University, Nanjing 210037, China; 4 Joint International Research Lab of Lignocellulosic Functional Materials, Nanjing Forestry University, Nanjing 210037, China

**Keywords:** nanoporous, MXene, cellulose nanofibers, ion transport, osmotic energy

## Abstract

Two-dimensional (2D) nanofluidic channels are emerging as potential candidates for harnessing osmotic energy from salinity gradients. However, conventional 2D nanofluidic membranes suffer from high transport resistance and low ion selectivity, leading to inefficient transport dynamics and limiting energy conversion performance. In this study, we present a novel composite membrane consisting of porous MXene (PMXene) nanosheets featuring etched nanopores, in conjunction with cellulose nanofibers (CNF), yielding enhancement in ion flux and ion selectivity. A mild H_2_O_2_ oxidant is employed to etch and perforate the MXene sheets to create a robust network of cation transportation nanochannels that effectively reduces the energy barrier for cation transport. Additionally, CNF with a unique nanosize and high charge density further enhances the charge density and mechanical stability of the nanofluidic system. Under neutral pH and room temperature, the PMXene/CNF membrane demonstrates a maximum output power density of 0.95 W·m^−2^ at a 50-fold KCl gradient. Notably, this represents a 43% improvement over the performance of the pristine MXene/CNF membrane. Moreover, 36 nanofluidic devices connected in series are demonstrated to achieve a stable voltage output of 5.27 V and power a calculator successfully. This work holds great promise for achieving sustainable energy harvesting with efficient osmotic energy conversion utilization.

## 1. Introduction

Global climate change has swiftly emerged as a pervasive threat that affects virtually every facet of our existence [1]. The primary driver behind this unprecedented climate transformation is widely acknowledged to be the burning of fossil fuels. Consequently, there has been a widespread and concerted effort to explore clean and sustainable energy sources as a means to meet the ever-growing global energy demands while concurrently minimizing the detrimental impacts on our environment [2]. One potential solution that has captured increasing attention in recent years is osmotic energy [3,4]. Osmotic energy offers a unique approach to addressing the global energy crisis. In systems like the osmotic energy generator, which relies on electrodialysis reversal (RED), the difference in salinity concentration propels ions in a single direction, thus generating ionic currents. Notably, this ion transfer-based energy conversion system possesses several advantages, including safety, reliability, and a simple energy conversion process, when compared to systems reliant on electron transfer [5]. Furthermore, a remarkable source of inspiration for ion transport-based osmotic energy generation has emerged from the natural world. Electric eels, in particular, have been found to possess sub-nanometer protein ion channels that enable them to harness electrical energy from ion gradients, resulting in astonishing voltage outputs of up to 600 V [6]. This discovery has sparked a wave of enthusiasm among researchers and innovators, driving forward the ongoing exploration and development of ion transport-based osmotic energy generators.

To date, a variety of one-dimensional (1D) nanofluidic materials have been developed for investigating ion transport and achieving remarkable power density outputs. These materials include PET conical nanopores [7], boron nitride nanotubes [8], and MoS_2_ nanopores [9]. While these materials have yielded surprising results in terms of energy acquisition, their intricate fabrication processes and high production costs have acted as significant barriers [5].

Recently, the realm of large-scale integration of nanofluidic channels has entered a transformative phase. Two-dimensional (2D) lamellar membranes, characterized by their superior ion selectivity and high ion flux, have emerged as a viable and scalable alternative for osmotic energy harvesting. These membranes feature slit-shaped 2D nanochannels nestled between neighboring nanosheets, creating sub-nanometer fluidic channels that facilitate ion diffusion guided by surface charges. This phenomenon is notably demonstrated by a range of 2D nanomaterials, including graphene oxide (GO) [10,11,12], carbon nitride [13,14], boron nitride [15,16,17], and vermiculite [18]. Nevertheless, nanochannels established through stacked non-porous 2D nanosheets often permit ions to flow inertially and vertically around sheet edges or junctions [19,20]. This results in an elongation of ion diffusion pathways and, consequently, slower ion transport. Hence, the strategic introduction of porous nanosheets becomes pivotal in affording a more intricate network of nanochannels. Such porous nanosheets effectively truncate ion transport routes, thereby expediting ion movement.

In the realm of energy harvesting, traditional 2D nanofluidic materials have seen remarkable advancements, with MXene, an innovative transition metal carbide, nitride, or hybrid thereof, standing out due to its layered structure and the presence of terminal functional groups (−Cl, −F, and −OH) on the nanosheet surface [21,22,23]. It has been reported that simply stacked films of 2D materials with insufficient charge density, such as graphene oxide or MoS_2_, exhibit ion transport kinetics that are monotonically controlled by the surface charge, with power densities below 1 W m^−2^ [14,24,25,26]. As a novel class of 2D layered transition metal carbides and nitrides, MXene has received increasing attention because of its high surface charge density due to the polar functional groups on its surface (-F, -OH, -O) and high conductivity with reduced transport resistance [27]. Notably, when MXene nanosheets are subjected to nanopore etching, the resulting etched holes with surface functional groups can be ingeniously utilized as pathways for cation transport. This innovative approach preserves ion selectivity while further enhancing osmotic power density [28]. Chemical vapor deposition (CVD) has certainly made it possible to prepare 2D materials such as GO [29], MoS_2_ [30], WS_2_ [31], etc. on a large scale and at low cost. Hao et al. grew graphene oxide sheets on carbon nanofibres (CNFs) by CVD [32], while Professor Dmitri V. Talapin’s team demonstrated a new strategy for the scalable and economical preparation of MXene by synthesising Ti_2_CCl_2_ and Ti_2_NCl_2_ MXenes at high temperatures via titanium metal, titanium chloride, and various carbon or nitrogen sources [33]. Existing studies show that the scale-up of MXene preparation by CVD is becoming a reality. The MXene nanoflakes were first lightly etched with H_2_O_2_ to produce TiO_2_ nanoparticles (NPs) embedded in situ on the surface of the MXene flakes. As the etching time increases, the TiO_2_ NPs grow along the initial nucleation sites, slowly ‘digging’ down and eventually ‘digging’ through the sandwich structure of the MXene nanoflakes, forming a large number of permeable pores on the surface of the MXene nanoflakes [34]. Simultaneously, cellulose nanofibers (CNF), derived from one of the most abundant natural materials, have garnered widespread interest in the realm of osmotic energy conversion. Their unique nanoscale dimensions, abundant modifiable functional groups, and excellent mechanical properties [35,36]. Various CNF-based composite nanofluidic membranes, such as GO/CNF [37,38,39,40], MXene/CNF [41,42], carbon nitride/CNF [13], and MoS_2_/CNF [43] membranes, have been designed and demonstrated to enhance the ion selectivity and chemical stability of 2D nanofluidic system, thereby facilitating high-efficiency osmotic energy conversion.

**Figure 1:**
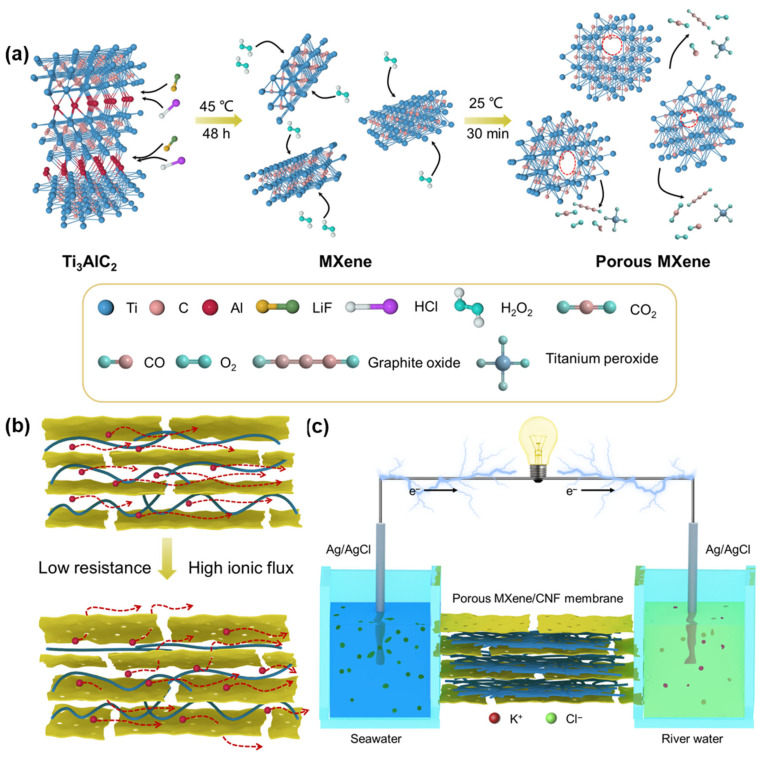
(**a**) Schematic representation of the preparation process for porous MXene nanosheets. (**b**) Comparative analysis between pristine MXene/CNF membranes and porous MXene/CNF composite membranes demonstrating increased ion transport channels, reduced internal resistance, and enhanced ion flux. (**c**) Application of porous MXene/CNF membrane-based nanofluidic devices for osmotic energy capture and its efficient conversion into electrical energy for output.

In this work, we present a composite membrane engineered by reassembling porous MXene (PMXene) nanosheets and CNF, establishing a robust platform for high-efficiency osmotic energy conversion. The nanopores within the MXene sheets are incorporated using a facile H_2_O_2_-based etching method (see Figure 1a). Importantly, the utilization of mild oxidizing agents ensures that the surface functionality of pristine MXene material remains intact. In comparison to the pristine MXene/CNF membrane (MCM), the etched nanoporous MXene/CNF composite membrane demonstrates superior energy conversion efficiency, enhanced ion selectivity, and a significantly reduced ion energy barrier (see Figure 1b). Furthermore, the energy conversion performance of these composite membranes can be fine-tuned by adjusting the nanofiber content and the electrolyte pH. Specifically, PMXene/CNF membrane containing 50 wt% CNF content achieves an impressive output power density of 1.29 W·m^−2^ at pH 9. This result underscores the positive impact of the PMXene and CNF composite approach on ion transport behavior. In a remarkable development, when 36 PMXene/CNF composite nanofluidic devices are connected in series, an output voltage of up to 5.26 V is attained, sufficient to power a calculator. This work exemplifies a viable path toward achieving high-efficiency osmotic energy conversion through porous materials and CNF-based nanofluidic membranes (Figure 1c).

## 2. Results

### 2.1. The Characteristics of Porous MXene/CNF Composite Membrane

**Figure 2:**
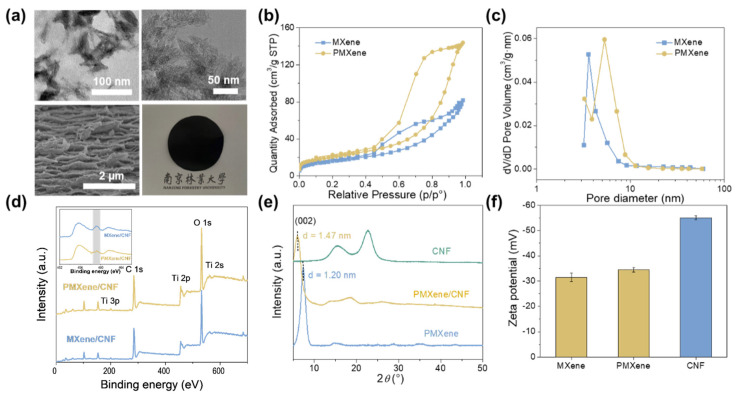
The characteristics of porous MXene/CNF composite membrane (PMCM). (**a**) TEM images of MXene and PMXene nanosheets, cross-section SEM image, and a photograph of PMCM. (**b**) Adsorption and desorption curves of MXene and PMXene. (**c**) Pore size distribution curves of MXene and PMXene. (**d**) XPS survey spectrum of PMXene sheets with etched pores. (**e**) XRD patterns of PMXene, PMCM, and CNF membranes. The interlayer spacing increases from 1.20 nm to 1.47 nm with the intercalation of nanofibers. (**f**) Zeta potential of MXene, PMXene, and CNF.

As previously reported, MXene nanosheets were synthesized by selectively etching Al atoms from the MAX phase Ti_3_AlC_2_ using LiF/HCl etching agents [44,45,46]. Subsequently, porous MXene (PMXene) sheets with uniformly distributed nanopores were obtained by mixing the aqueous suspension of the synthesized Ti_3_C_2_T_x_ sheets with an H_2_O_2_ solution at a specific volume ratio (see Figure 1a) [47]. As depicted in Figure 2a, the majority of the etched MXene forms multi-layered nanosheet stacking structures. Further treatment with H_2_O_2_ etching is evident in the TEM image, which reveals that the PMXene transitions into a monolayer nanosheet structure with abundant well-defined etched nanopores. The TEM image shows that CNF exhibited a homogeneous single-fiber morphology with an average diameter of 18.04 nm (see Figure S1). Free-standing membranes composed of PMXene and CNF were fabricated using a vacuum suction filtration assembly. From the SEM images of the cross section, it is apparent that the PMXene/CNF composite membrane (PMCM) features abundant layered nanochannels conducive to ion transport. Meanwhile, the smooth surface of the PMXene/CNF membrane suggests that the PMXene sheets did not introduce any significant defects (see Figure S2). The Brunauer-Emmett-Teller (BET) surface area and pore size distribution of MXene and PMXene were investigated using N_2_ adsorption–desorption isotherms (see Figure 2b,c). The BET surface area of PMXene (73.39 m^2^/g) surpasses that of MXene (57.21 m^2^/g), while the average diameter of the etched nanopores measures is 3.2 nm. Obviously, H_2_O_2_ etching enhances the porosity of PMXene, facilitating unobstructed channels for ion diffusion and improving the energy conversion performance. Additionally, the chemical composition of the synthesized MXene and PMXene was examined using X-ray photoelectron spectroscopy (XPS), as depicted in Figure 2d. High-resolution XPS spectra of the Ti2p core levels of pristine MXene and etched PMXene, inset in Figure 2d, demonstrate that the H_2_O_2_-induced oxidation effect is minimal, leading to the formation of a small amount of TiO_2_ compared to pristine MXene. The XPS spectra confirm the presence of abundant functional groups on the surface of both pristine MXene and PMXene nanosheets. Furthermore, the porous modification of MXene nanosheets increases their hydrophilicity. The PMXene/CNF composite strategy further reduces the contact angle, indicating an additional enhancement in hydrophilicity (see Figure S3). Simultaneously, the introduction of CNF between PMXene nanosheets widens the interlayer spacing and reduces ion transport resistance. As shown in Figure 2e, the addition of CNF increases the PMXene membrane layer spacing from 1.2 nm to 1.47 nm. Moreover, the surface charge density of PMXene and CNF was determined via zeta potential (see Figure 2f). Compared to pristine MXene, H_2_O_2_ etching does not result in any surface charge loss in PMXene. Meanwhile, CNF exhibits a significantly higher potential (−55.12 mV) than MXene (−31.69 mV) and PMXene (−34.37 mV) due to its abundance of carboxyl groups.

### 2.2. Ion Transport Properties of the PMCM

**Figure 3:**
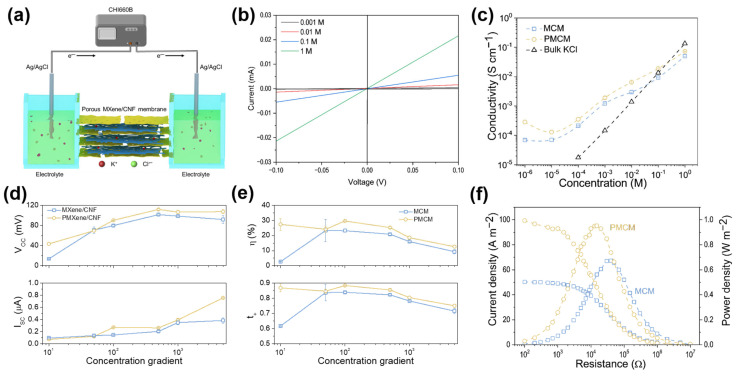
(**a**) Schematic representation of the ion transport device. (**b**) I-V curves of PMCM at different KCl concentrations. (**c**) Ionic conductivity of PMCM and MCM at varying KCl concentrations. (**d**) Comparative osmotic current and potential of PMCM and MCM under concentration gradient conditions (CL = 0.1 mM, CH = 1 mM–0.5 M). (**e**) Energy conversion efficiency and cation selectivity across different KCl concentration gradients. (**f**) Power density and current density are plotted as a function of external resistance.

To examine the impact of etched nanopores on ion transport in composite membranes, we compared the ionic conductivity and current–voltage (I-V) characteristics of MCM and PMCM across varying KCl concentrations and concentration gradients. The transmembrane I-V curve was assessed using the experimental setup shown in Figure 3a. The composite membrane was positioned between two electrolytic cells, featuring an ion transport area of 0.03 mm^2^. Through the laminar nanochannels of the composite membrane, ions in the electrolyte underwent selective transport, while a pair of standard Ag/AgCl electrodes gauged transmembrane current and voltage. Figure S4 illustrates that the I-V curves of PMCM and MCM comply with Ohm’s law. As electrolyte concentration rises, the current values at +0.1 V also ascend, indicative of an elevated slope in I-V curves (see Figure 3b). According to Equation (S1), the ionic conductivity of composite membranes at different KCl concentrations was calculated (see Figure 3c). In bulk salt solutions, ionic conductivity aligns linearly with salt concentration (black dashed line) [42].

The ion transport driven by concentration gradient across the composite membrane was further assessed. Open-circuit membrane voltage (V_OC_) and short-circuit current (I_SC_) were recorded when the membrane was exposed to an asymmetric KCl electrolyte gradient. Due to the cation selectivity of PMCM, cations spontaneously diffuse preferentially along the nanochannels from the high to the low concentration side. This diffusion generates a transmembrane concentration gradient in both current and potential. Figure 3d displays V_OC_ and I_SC_ for PMCM and MCM under a series of KCl electrolyte concentration gradients, spanning from low concentrations of 10^−4^ M to high concentrations up to 0.5 M. As the concentration gradient increased from 10-fold to 5000-fold, V_OC_ rose from 43.38 to 107.52 mV, and I_SC_ increased from 0.10 to 0.76 μA. Using Equations (S2) and (S3) from the SI, we calculated the cation selectivity and energy conversion efficiency of the two composite membranes, as shown in Figure 3e. PMCM demonstrated a maximum energy conversion efficiency of 29.61% and a maximum cation selectivity of 0.89, both exceeding those of MCM (24.40% and 0.84). Notably, both energy conversion efficiency and cation selectivity of the composite membrane decreased beyond the 100-fold concentration gradient due to the reduction in double electric layer thickness under increased electrolyte concentration [48]. Additionally, we also explored the relationship between current density, power density, and external resistance for both composite membranes under a 50-fold concentration gradient in artificial seawater (0.5 M KCl) and river water (0.01 M KCl). Output power was calculated directly using the equation P = I^2^R_L_, where I is the measurement current, and R_L_ is the external resistance [42]. When the external resistance equals the internal resistance of the membrane, the extractable power reaches its maximum. Figure 3f shows that with R_L_ at 37 kΩ, MCM achieved a maximum output power density of 0.67 W·m^−2^. In contrast, PMCM exhibited a reduced internal resistance of 14 kΩ, coupled with an increased maximum output power density of 0.95 W·m^−2^, confirming that the nanopore PMXene preparation strategy effectively lowered the internal resistance of nanofluidic membrane transport and improved its energy conversion performance. Furthermore, PMCM demonstrated excellent operational stability, maintaining a power density of up to 0.86 W·m^−2^ after 9 days of continuous operation (Figure S5).

**Figure 4:**
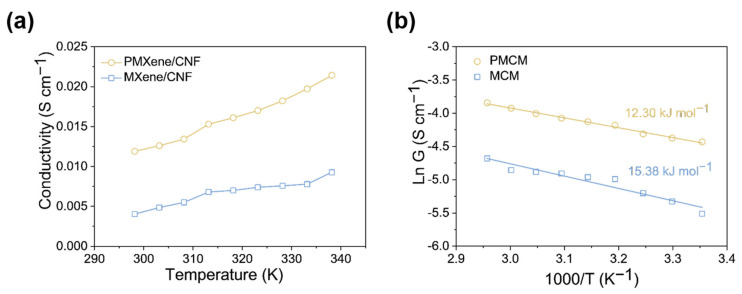
(**a**) Ionic conductance of PMCM and MCM at various temperatures using a 10^−2^ M KCl electrolyte. (**b**) Arrhenius plot displaying the logarithmic conductance versus inverse temperature derived from PMCM and MCM.

Additionally, we calculated the K^+^ transport energy barrier to assess the impact of etched nanopores on osmotic energy conversion. As shown in Figure 4a, both PMCM and MCM exhibit ionic conductance that is linearly dependent on temperature within the range of 278–333 K, following Arrhenius’s behavior. The temperature dependence of the membrane is attributed to increased ion mobility as fluid viscosity decreases [49]. The K^+^ transport barrier within the composite membranes was calculated using the Arrhenius equation [50]:

$$G=G_{0}e^{\frac{-\mathrm{Ea}}{\mathrm{RT}}},$$

where G represents conductance, G_0_ is a constant, R is the gas constant, T is the temperature, and Ea is the activation energy. The energy barrier of K+ transport across the stacked PMCM nanochannels measures 12.3 kJ·mol^−1^, which is lower than that of the pristine MCM at 15.38 kJ·mol^−1^ (see Figure 4b). This indicates that the reduced energy barrier in PMCM with etched nanopores results in enhanced osmotic diffusion.

### 2.3. The Regulation of PMCM Energy Conversion Properties

**Figure 5:**
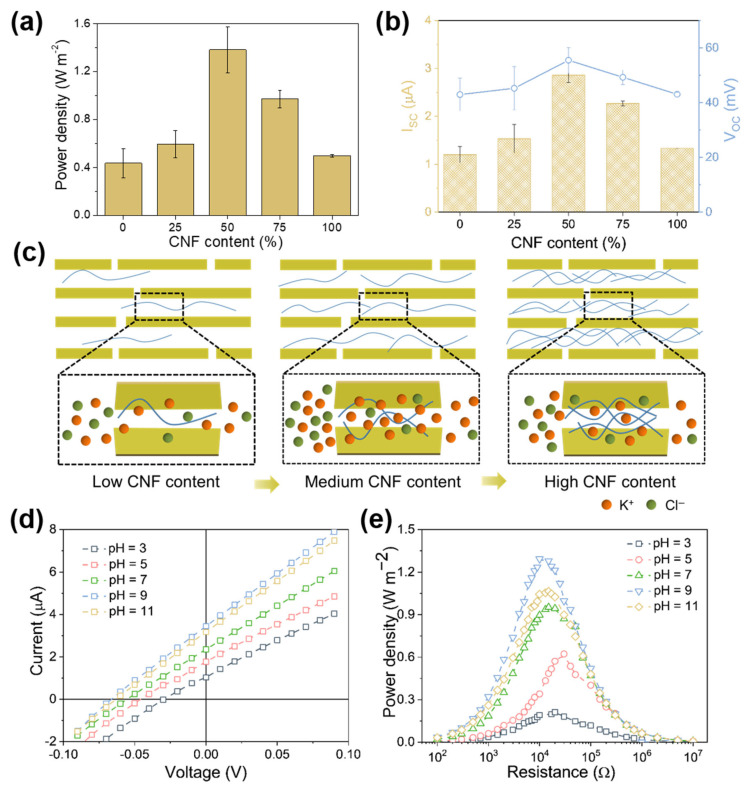
(**a**) The output power density of PMXene/CNF membranes at varying CNF content. (**b**) Impact of CNF content on the values of V_OC_ and I_SC_ for PMCMs. (**c**) Schematic illustrating the influence of CNF content on the energy conversion process. (**d**) I-V curves of PMCMs under a 50-fold concentration gradient (C_H_ = 0.5 M KCl, C_L_ = 0.01 M KCl) and at different pH conditions. (**e**) The output power density of PMCM under a 50-fold concentration gradient and at different pH conditions.

Furthermore, we investigated the impact of CNF content on the osmotic energy conversion performance of PMCM while maintaining a 50-fold concentration gradient (0.5 M and 0.01 M KCl). The maximum output power (P_m_) was calculated from V_OC_ and I_SC_ using the equation P_m_ = V_OC_ × I_SC_/4 [13]. As shown in Figure 5a, the weight content of CNF (ranging from 0 to 100%) significantly influences energy conversion performance. Power density initially increases from 0.60 W·m^−2^ to 1.38 W·m^−2^ when CNF content reaches 50%. However, as CNF content rises to 75%, power density decreases to 0.94 W·m^−2^. Notably, the PMXene/CNF composite strategy notably enhances osmotic energy conversion performance compared to both PMXene membrane and CNF membrane across all CNF content ranges. To gain further insights into the mechanism behind the increased power density in the composite strategy, we examined V_OC_ and I_SC_ across varying CNF content, as shown in Figure 5b. Due to its high ion selectivity, pure PMXene and CNF membranes attain higher V_OC_ values. However, their lower ion flux and high ion transport energy barrier lead to lower I_SC_ values. As CNF content increases, V_OC_ initially rises from 42.97 to 68 mV and then decreases to 50.91 mV at 75% CNF content. Correspondingly, I_SC_ peaks at 50% CNF content (2.86 μA), which is 2.4 times that of the pure PMXene membrane and 2.1 times that of the CNF membrane. Although I_SC_ decreases at high CNF content (75%), the output power density remains higher than that of the pure PMXene and CNF membrane.

To further investigate the effect of CNF content on ion transport behavior, we calculated the cation transport number (t_+_) using Equation (S2). As shown in Figure S6, the t_+_ value for the pure PMXene membrane was 0.73, which was higher due to the confined space between the PMXene nanosheets. A high t_+_ value of 0.79 for the composite membranes indicates that PMCM exhibits good cation selectivity. These results highlight that the critical CNF content for optimal power density is 50%. Around this optimal point, CNF intercalation expands the interlayer distance between PMXene nanosheets, enhancing ion flux through the nanochannels (see Figure 5c). Simultaneously, CNF enhances the surface charge density of the composite system while maintaining high ion selectivity in the expanded nanochannels (see Figure S7). However, above this critical CNF content, the salinity gradient energy harvesting performance is disrupted. This occurs because excess CNF partially obstructs the 2-D channels and increases spatial site resistance to ion transport (see Figure 5c). This phenomenon is supported by cross-sectional SEM images of PMCM with varying CNF contents (see Figure S8). Consequently, excess CNF decreases ion flux, thereby weakening output power density.

Since the energy conversion mainly depends on the surface charge of PMXene and CNF, the output power density of the PMXene/CNF membrane is strongly influenced by the electrolyte pH. As the electrolyte pH rises from 3 to 11, both V_OC_ and I_SC_ simultaneously increase, eventually reaching 66 mV and 3.46 μA (see Figure 5d), respectively. The composite membrane’s output power density also increases, peaking at 1.29 W·m^−2^ at pH 9 (see Figure 5e). This phenomenon arises because higher pH values lead to the deprotonation of the –OH groups and –COOH groups carried by PMXene and CNF, resulting in an increased surface charge density of the composite system. As shown in Figure S9, as pH increases from 3 to 11, the zeta potential of the composite system increases from −30.66 to −41.47 mV.

**Figure 6:**
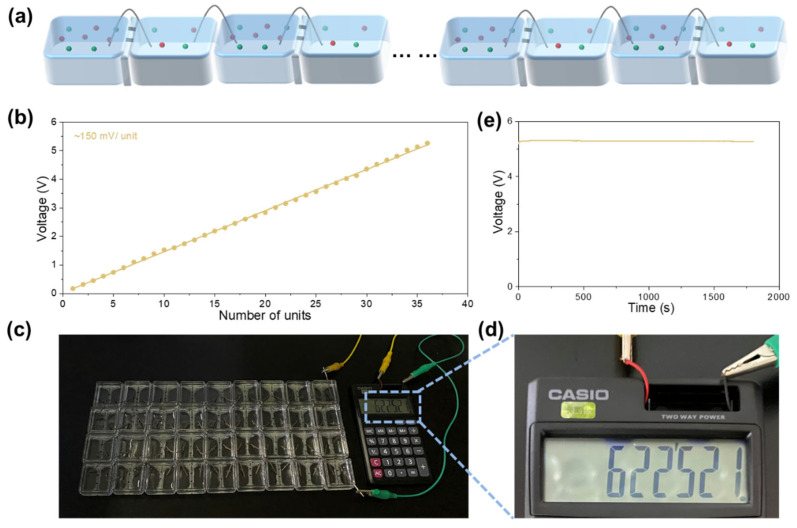
(**a**) Schematic illustration of the PMCM-based nanofluidic devices connected in series. (**b**) Increase in output voltage with the number of units under a 50-fold concentration gradient. (**c**) Photograph displaying an array of 36 units of PMCM-based nanofluidic devices, utilized to power a calculator. (**d**) Enlarged view of the calculator powered by the nanofluidic device. (**e**) PMCM-based nanofluidic devices maintain a stable output voltage of 5.27 V over a duration of 1800 s.

To illustrate the suitability of PMXene/CNF composite membranes as nanofluidic devices for osmotic energy harvesting and power generation, 36 identical devices were prepared, encompassing a total cross-sectional area of 0.06 mm^2^. These devices were interlinked via Ag/AgCl electrodes, as shown in Figure 6a. The output voltage, as shown in Figure 6b, reached 5.26 V within a 50-fold concentration gradient (0.01 and 0.5 M KCl), with an average voltage of 150 mV. Subsequently, the nanofluidic device demonstrated its capability to power a calculator and perform arithmetic operations under the same 50-fold concentration gradient conditions (see Figure 6c,d). Notably, the nanofluidic device exhibited long-term continuous and stable operation. The PMXene/CNF composite membrane-based osmotic energy generator consistently and steadily produced an output voltage of up to 5.27 V over a duration of 1800 s (Figure 6e). Additionally, the incorporation of CNF significantly enhanced the mechanical properties of PMCM. With a CNF content of 50%, PMCM achieved a maximum tensile strength of 165.9 MPa, making it well-suited for practical applications in osmotic energy generation (Figure S10).

## 3. Discussion

PMCM and MCM manifest distinct behaviors in terms of ionic conductivity. This can be explained by the fact that at high electrolyte concentrations, transmembrane ionic conductivity approximates that of bulk salt solutions. However, as electrolyte concentration decreases, composite membranes ionic conductivity progressively deviates from volumetric values, plateauing at low concentrations. Due to the negative charges on MXene, PMXene, and CNF, cations (e.g., K^+^) accumulate within the channel while anions (e.g., Cl^−^) are excluded, yielding a unipolar cation solution within the channel. In such cases, surface charge governs ion transport within the channel, resulting in higher ionic conductivity than bulk solutions at low concentrations [51]. Importantly, PMCM consistently exhibits higher conductivity than MCM, confirming that etched nanopores effectively enhance K+ flux, thereby elevating conductivity.

## 4. Materials and Methods

### 4.1. Materials

Ti_3_AlC_2_ MAX phase (400 mesh) was acquired from 11 Technology Co., Ltd. (Changchun, China). Cellulose nanofiber (CNF, 1.07 wt%) was procured from Tianjin Wood Spirit Biotechnology Co., Ltd. (Tianjin, China). Lithium fluoride (LiF) was obtained from Shanghai Aladdin Biochemical Technology Co., Ltd. (Shanghai, China). Hydrochloric acid (HCl), hydrogen peroxide (H_2_O_2_), potassium chloride (KCl), sodium chloride (NaCl), magnesium chloride (MgCl), calcium chloride (CaCl), and sodium hydroxide (NaOH) were purchased from Sinopharm Chemical Reagent Co., Ltd. (Shanghai, China). A filter membrane (diameter ~50 mm, pore size ~0.22 µm) was obtained from Shanghai Xinya Purification Device Factory (Shanghai, China). All materials were employed in their original states without any modifications.

### 4.2. Preparation of TEMPO Oxidised CNF

CNF was prepared from softwood bleached kraft pulp according to Isogai [52] et al. 300 mL of deionized water was placed in a beaker, 3 g of adiabatic softwood pulp, 0.045 g of TEMPO and 0.3 g of NaBr were added and the mixture was stirred on a magnetic stirrer for 10 min. After adding the required amount of 10 mmol g^−1^ NaClO solution, the pH of the mixture was adjusted to 10 by using 9 mol L^−1^ aqueous HCl solution to adjust the pH of the mixture = 10 and the oxidation reaction was started. During the oxidation reaction, the pH of the suspension was maintained at 10 with continuous stirring at room temperature and adjusted with 0.5 mol L^−1^ aqueous NaOH solution. After 2 h, the oxidation reaction was stopped by adding 3 mL of anhydrous ethanol. After the reaction, the suspension was washed with de-ultrapure water to neutral (pH = 7). Finally, the transparent gel-like fiber solution was obtained by high-pressure homogenization with mechanical shear treatment.

### 4.3. Synthesis of Porous MXene Nanosheets

The synthesis of MXene (Ti_3_C_2_T_X_) was conducted using the LiF/HCl etching method, following a previously established protocol [32]. 1.6 g of LiF was stirred and mixed with 20 mL of 9 M HCl for 5 min to obtain the etchant solution. Then 1.0 g Ti_3_AlC_2_ powder was slowly added and magnetically stirred at 35 °C for 48 h to complete etching. The suspension was then washed with ultrapure water at 3500 rpm for 5 min until the pH of the supernatant was ≥6. The purified suspension was sonicated for 20 min to disperse the MXene nanoparticles. Finally, the impurities were removed by centrifugation at 3500 rpm for 5 min to obtain a well-dispersed MXene suspension. The fabrication of porous MXene nanosheets was carried out according to a method previously reported by Liu et al. [34]. Firstly, 10 mL of 5 mg mL^−1^ MXene suspension and 90 mL of 1 μM H_2_O_2_ solution (1:9, *v*/*v*) were added to a beaker and homogeneously mixed, and then the reaction was left at room temperature for 30 min. After the reaction, the supernatant was washed with ultrapure water by centrifugation at 8000 rpm for 15 min, and the UV spectrum of the supernatant showed no TiO_2_ absorption peak. Finally, the PMXene solution was sonicated for 20 min to obtain uniformly dispersed PMXene nanosheets.

### 4.4. Preparation of Porous MXene/CNF Composite Membrane

The PMXene solution and the gel-like CNF were dispersed in ultrapure water to form a 1 mg mL^−1^ solution. The absolute dry mass of the composite membrane was controlled to be 40 mg and the two solutions were mixed according to the percentage of absolute dry mass (CNF mass fraction) of 25%, 50% and 75%, then stirred for 30 min and sonicated for 20 min. The mixed solutions were vacuum-evacuated on a composite cellulose filter membrane (50 mm diameter, 0.22 μm pore size) and washed twice with 10 mL of ultrapure water. The composite membrane was dried at room temperature for 24 h before being peeled off the substrate.

### 4.5. Electrochemical Measurement

An electrochemical workstation and a Keithley 2400 digital multimeter were used to test the ion transport and salt differential energy conversion properties of the composite membranes. Membrane samples were placed in a custom-designed two-compartment polydimethylsiloxane (PDMS) electrochemical setup and transmembrane potentials were measured using standard Ag/AgCl electrodes. Current–voltage (I-V) curves of the PMXene/CNF membranes were recorded over a range of electrolyte concentrations from 10^−6^ to 1 M and voltages from −1 to +1 V. Meanwhile, the conductivity of a series of KCl solutions of known concentration was evaluated using a commercial conductivity meter to establish a reference curve for KCl. Current–voltage curves at different pH values were tested by adjusting the pH of the electrolyte. The samples were immersed in electrolyte for more than 24 h prior to each measurement to ensure the accuracy and reliability of the test results.

### 4.6. Characterization

To thoroughly understand the properties and structure of the materials involved, a range of characterization techniques were employed.

The morphological structure of the cross-section and surface was analyzed using a scanning electron microscope (SEM, JSM-7600F, JEOL, Shimazu, Tokyo, Japan) after the samples had been gold-sprayed.

The morphology and microstructure of the samples were observed using a high-resolution transmission electron microscope (HRTEM, JEM-1400, JEOL, Tokyo, Japan) after the PMXene dispersion was diluted to 0.1 mg mL^−1^ with anhydrous ethanol.

The samples were scanned using an atomic force microscope (Dimension Edge, Bruker, Karlsruhe, Germany) in tapping mode and later analyzed for the thickness of the MXene nanosheets and the diameter of the CNFs using NanoScope Analysis software (v.1.40r3sr2). The elemental composition and chemical states were analyzed using an X-ray photoelectron spectrometer (AXIS Ultra DLD, Shimadzu, Kyoto, Japan).

The crystalline structures were studied using XRD patterns recorded on an X-ray diffractometer (Ultima IV diffractometer, Rigaku, Tokyo, Japan) with Cu Kɑ radiation (λ = 0.154 nm) operated at 40 kV and 40 mA.

Zeta potentials of MXene and CNF were determined using a Zeta potentiometer (Zetasizer Nano-Zs, Malvern, Worcestershire, UK) with stable 1 mg·mL^−1^ suspensions. The pH adjustments were performed using 0.1 mol·L^−1^ HCl and 0.1 mol·L^−1^ KOH aqueous solutions.

The water contact angle of the composite membranes was measured using a contact angle meter (T200-Auto3 Plus, Biolin Scientific, Lund, Sweden).

For Brunauer–Emmett–Teller (BET) analysis, MXene and porous MXene samples were degassed with nitrogen at 120 °C for 6 h. BET parameters were measured and analyzed using a specific surface area and porosity analyzer (ASAP 2460, Micromeritics instruments, Norcross, GA, USA).

The mechanical properties of the composite membrane were evaluated using a tensile testing machine (AGS-X 500N, Shimadzu, Kyoto, Japan) at a loading rate of 5 mm·min^−1^. Tensile strengths were derived from the stress-strain curves, while toughness was calculated based on the area under the curves. All reported tensile strengths and toughness values were averaged from three samples.

## 5. Conclusions

In summary, we have successfully developed PMXene/CNF composite nanofluidic membranes and demonstrated their efficacy in achieving high-performance osmotic power generation. Through controlled etching with the mild oxidizer H_2_O_2_, we created nanopores with a diameter of 3.2 nm on MXene sheets. These etched nanopores function as interconnected cationic nanochannels, thereby significantly enhancing ion flux and cation selectivity within the composite system. Simultaneously, the inclusion of CNF further enhances the energy conversion performance by increasing the constricted nanochannels and charge density. The power density of the membrane can be finely tuned by adjusting the CNF content. Notably, when the CNF content is set at 50%, and the KCl concentration gradient is maintained at 0.5 M/0.01 M, the power density peaks at an impressive 0.95 W·m^−2^. By fine-tuning the pH value of the electrolyte, we can further boost the power density to reach an impressive 1.29 W·m^−2^. Moreover, when 36 PMXene/CNF components are connected in series, they demonstrate exceptional stability, producing a consistent voltage output of 5.26 V, capable of powering devices such as calculators over an extended period. This research represents a significant contribution to the advancement of composite membranes based on 2D nanofluidic materials and CNF in the field of sustainable green energy conversion, paving the way for innovative developments in this area.

## Data Availability

Data is contained within the article and Supplementary Materials.

## Supplementary Materials

The following supporting information can be downloaded at: https://www.mdpi.com/article/10.3390/ijms252313226/s1. References [24,53,54] are cited in the Supplementary Materials.
