# Supplementary material for: Fabrication of Porous MXene/Cellulose Nanofibers Composite Membrane for Maximum Osmotic Energy Harvesting
Source: Int J Mol Sci. 2024 Dec 9;25(23):13226. doi: 10.3390/ijms252313226 (PMC11642219; doi:10.3390/ijms252313226)
Supplement: Supplementary file 1 [file ijms-25-13226-s001.zip › ijms-3351771-supplementary.pdf]

Supporting Information

Fabrication of Porous MXene/Cellulose Nanofibers Composite Membrane for Maximum Osmotic Energy Harvesting

Sha Wang [1,2,*], Zhe Sun [1], Mehraj Ahmad [3,4] and Mengyu Miao [1]

[1] Jiangsu Co-Innovation Center of Efficient Processing and Utilization of Forest Resources, Nanjing Forestry University, Nanjing 210037, China

[2] International Innovation Center for Forest Chemicals and Materials, Nanjing Forestry University, Nanjing 210037, China

[3] Department of Food Science and Engineering, College of Light Industry and Food, Nanjing Forestry University, Nanjing 210037, China

[4] Joint International Research Lab of Lignocellulosic Functional Materials, Nanjing Forestry University, Nanjing 210037, China

* Correspondence: swang@njfu.edu.cn

1. Ionic conductivity of PMXene/CNF composite membrane

In the case of PMXene/CNF composite membrane, according to the Eq. (S1), the ionic conductivity can be calculated (Chen et al. 2019):

$$\lambda = l / SR \tag{S1}$$

where l, S, and R are the length, cross-sectional area, and the measured resistance in Eq. (S1).

2. Cation selectivity and energy Conversion Efficiency

The cation transference number under a specific concentration gradient can be calculated as (Zhang et al. 2019):

$$t_{+} = \frac{1}{2}\left(\frac{V_{OC}}{\frac{RT}{zF}\ln\left(\frac{r_{CH}CH}{r_{CL}CL}\right)} + 1\right) \tag{S2}$$

Herein, V_{OC}, R, T, F, z, r, and c refer to the open-current potential, universal gas constant, absolute temperature, Faraday constant, charge number, activity coefficient of ions, and ion concentration, respectively.

Energy conversion efficiency is defined as the ratio of the electrical energy output to the gibbs free energy input, which can be calculated according to the following equation (Zhang et al. 2019):

$$\eta_{max} = \frac{(2t_{+}-1)^2}{2} \tag{S3}$$

3. Surface Charge Density

Then we can estimate the surface charge density according to the equation (S4) (Van Hai et al. 2019),

$$\sigma = \frac{\varepsilon\varepsilon_0\zeta}{\lambda_d} \tag{S4}$$

in which σ is the surface charge, ε is the dielectric constant, ε_0 is the permittivity of vacuum, λ_d is the Debye length, and ζ is the zeta potential in equation (S4).

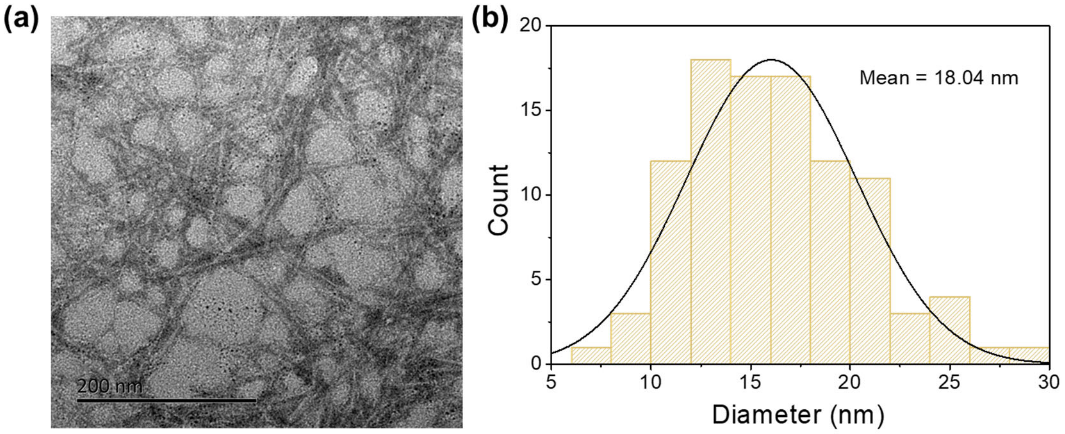

Figure S1. (a) TEM image of cellulose nanofibers (CNF). (b) The diameter analysis of CNF

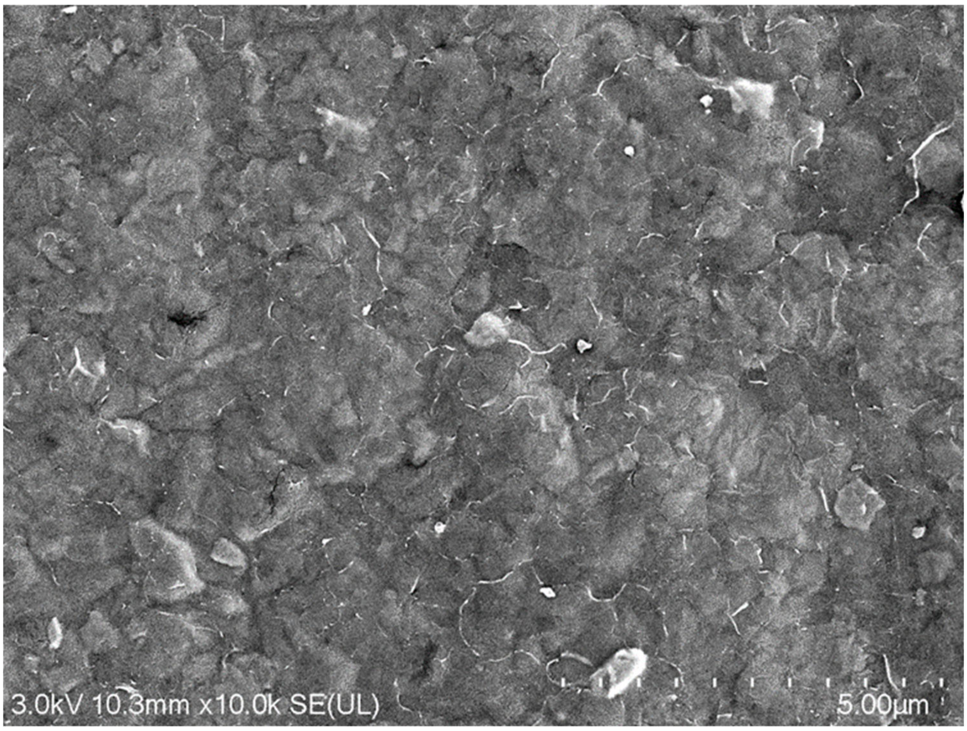

Figure S2. Surface SEM image of PMXene/CNF membrane

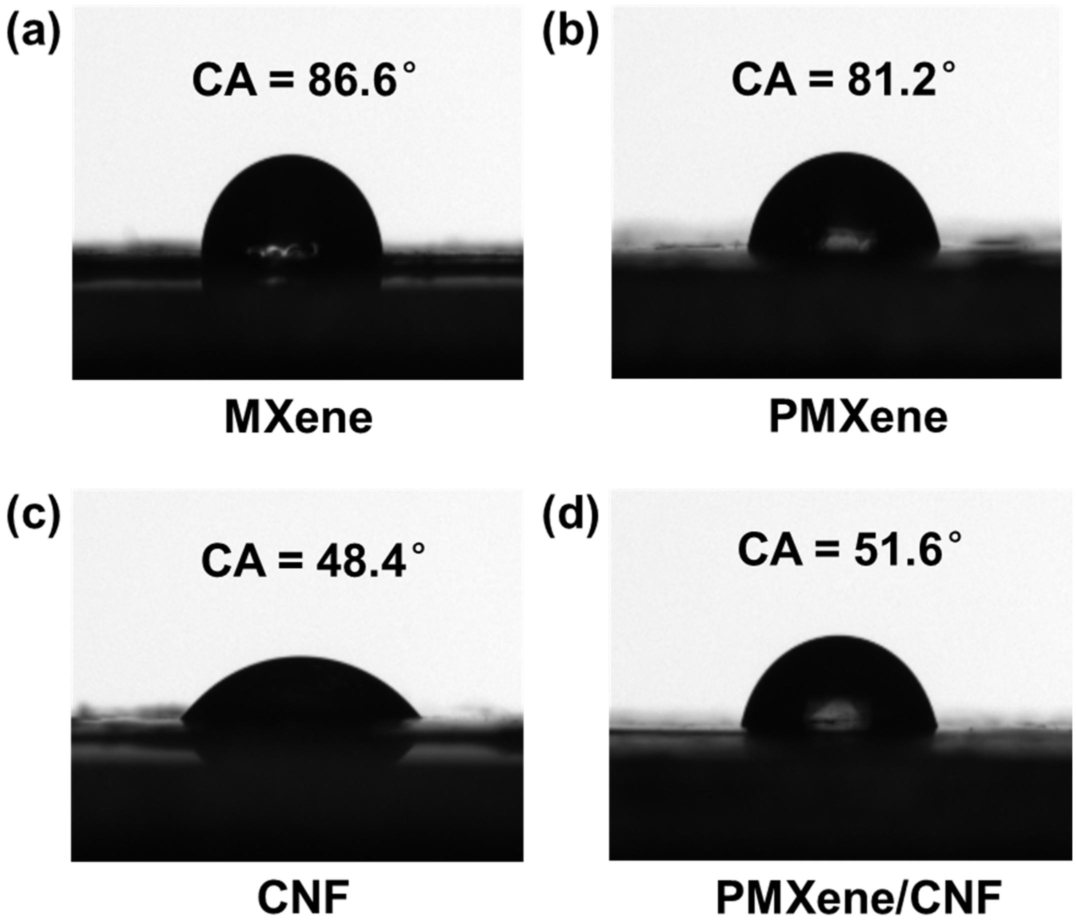

Figure S3. Contact angle of MXene (a), PMXene (b), CNF (c), PMXene/CNF (d) membranes

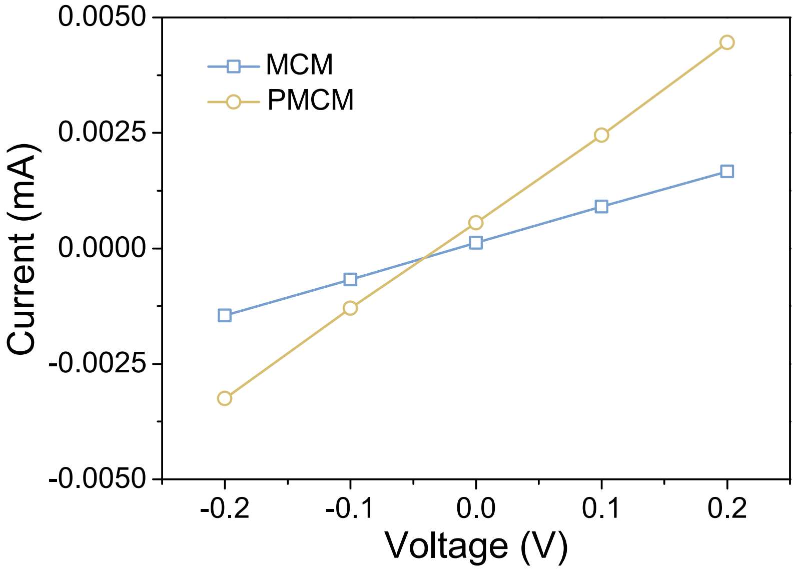

Figure S4. The I-V curves of MXene/CNF and PMXene/CNF composite membranes in 10^{-2} M KCl electrolyte

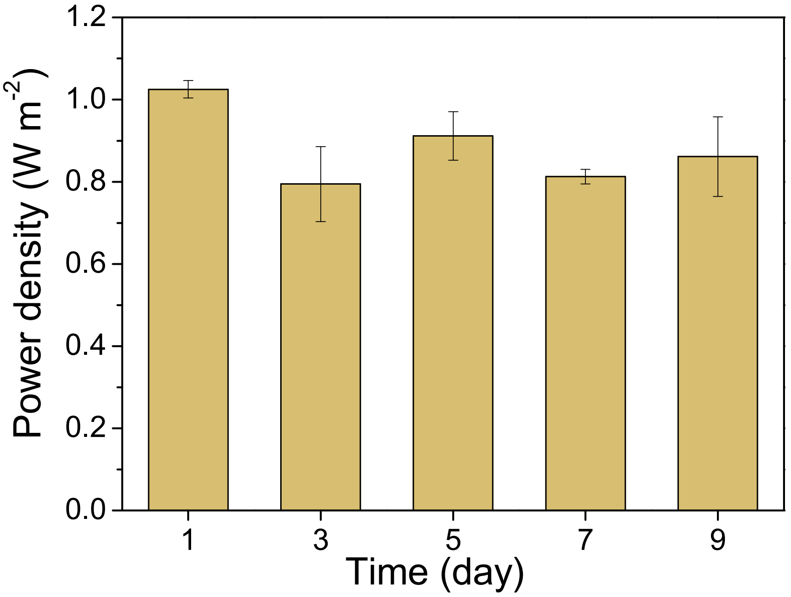

Figure S5. The stability of PMXene/CNF composite membranes

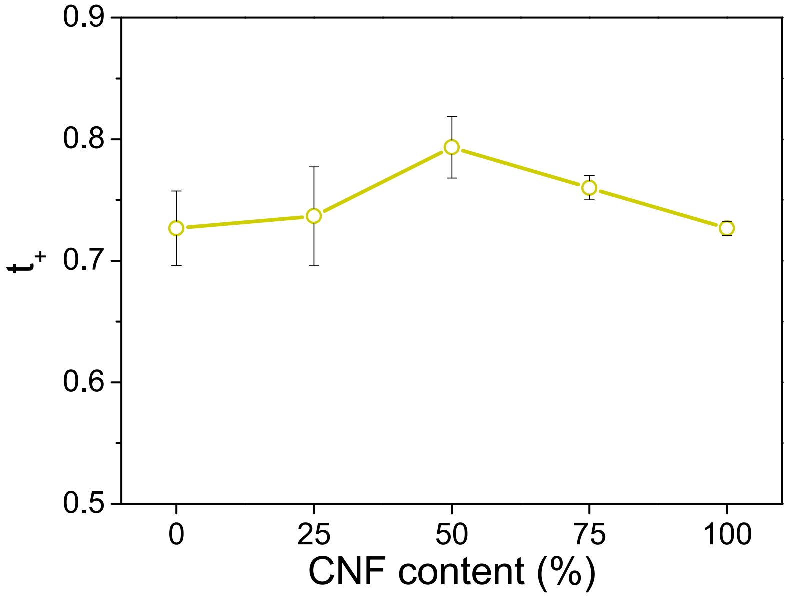

Figure S6. Cation transference number (t^+) of PMXene/CNF composite membranes with different CNF content

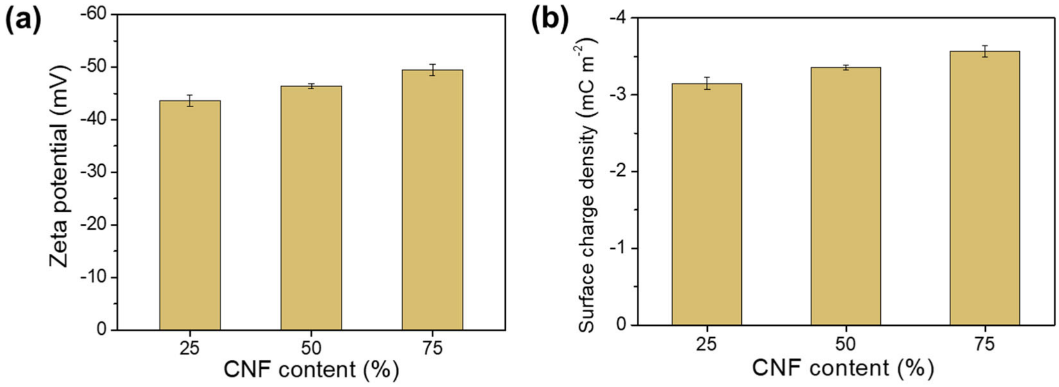

Figure S7. Zeta potential (a) and surface charge density (b) of PMXene/CNF composite membranes with different CNF contents

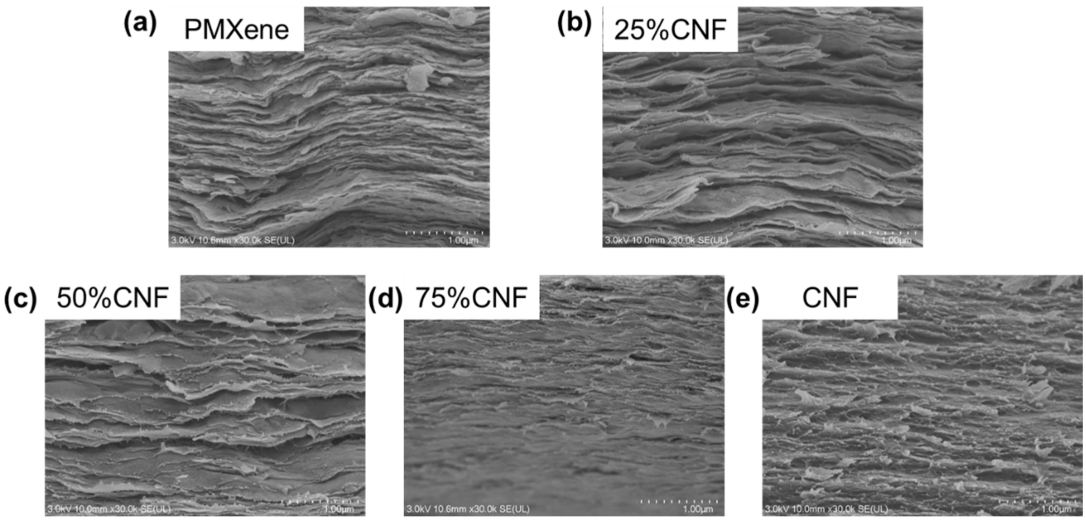

Figure S8. SEM images of PMXene/CNF composite membranes with different CNF contents

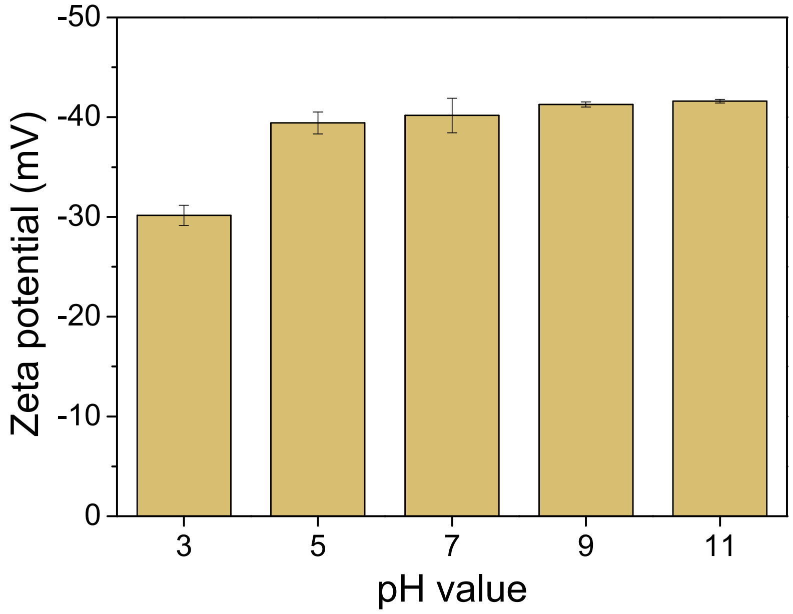

Figure S9. Zeta potential of PMXene/CNF composite membranes under different pH conditions

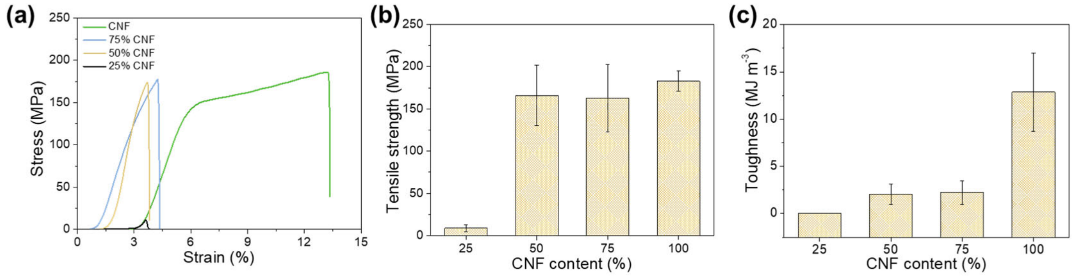

Figure S10. The mechanical performance of porous MXene/CNF membranes

References

Chen GG, Li T, Chen CJ, Wang CW, Liu Y, Kong WQ, Liu DP, Jiang B, He SM, Kuang YD, Hu LB (2019) A Highly Conductive Cationic Wood Membrane. Adv Funct Mater 29(44):1902772. http://doi.org.10.1002/adfm.201902772

Van Hai L, Zhai HC, Kim JW, Kim J (2019) Green Nanocomposites Made With Polyvinyl Alcohol and Cellulose Nanofibers Isolated From Recycled Paper. J Renew Mater 7(7): 621-629. http://doi.org.10.32604/jrm.2019.06466

Zhang Z, Yang S, Zhang PP, Zhang J, Chen GB, Feng XL (2019) Mechanically strong MXene/Kevlar nanofiber composite membranes as high-performance nanofluidic osmotic power generators. Nat Commun 10(1):2920. http://doi.org.10.1038/s41467-019-10885-8
